# Supplementary material for: Are Ethiopian diabetic patients protected from financial hardship?
Source: PLoS One. 2021 Jan 27;16(1):e0245839. doi: 10.1371/journal.pone.0245839 (PMC7840028; doi:10.1371/journal.pone.0245839)
Supplement: S1 Table — (DOCX) [file pone.0245839.s001.docx]

**S1 Table 1**: Clinical Characteristics of Diabetes Mellitus and Related issues among diabetic patients having regular follow up at public hospitals of Bahir Dar city administration, North West Ethiopia, 2019

| Characteristics | | Frequency | Percentage | mean | SD |
| --- | --- | --- | --- | --- | --- |
| Type of DM | Type 1 | 158 | 39.4 |  |  |
|  | Type2 | 243 | 60.6 |  |  |
| Duration of illness | <10 years | 323 | 80.5 | 7.72 | 5.43 |
|  | > 10 years | 78 | 19.5 |  |  |
| Frequency of follow up | Monthly | 158 | 39.4 |  |  |
|  | Two monthly | 69 | 17.2 |  |  |
|  | Three monthly | 112 | 27.9 |  |  |
|  | Four monthly | 62 | 15.5 |  |  |
| Degree of stress due to DM | Stressed | 287 | 71.6 |  |  |
|  | Not stressed | 114 | 28.4 |  |  |
| Prevention measures | Doing preventive measures | 170 | 42.4 |  |  |
|  | Not doing preventive measure | 231 | 57.6 |  |  |
