# Supplementary material for: Are Ethiopian diabetic patients protected from financial hardship?
Source: PLoS One. 2021 Jan 27;16(1):e0245839. doi: 10.1371/journal.pone.0245839 (PMC7840028; doi:10.1371/journal.pone.0245839)
Supplement: S2 Table — (DOC) [file pone.0245839.s002.doc]

S2 Table 2: Coping strategies for Diabetes Mellitus health care costs among diabetic patients having regular follow up at public hospitals of Bahir Dar city administration, North West Ethiopia, 2019

Coping mechanisms DM health care costs	
Coping mechanisms	Frequency	Percent	
Own money(salary, savings)	243	60.6	
Borrowing 	12	3.0	
Sell assets	54	13.5	
Family/relative support	85	21.2	
Others 	7	1.7	
Total	401	100.0	
