# Supplementary material for: Are Ethiopian diabetic patients protected from financial hardship?
Source: PLoS One. 2021 Jan 27;16(1):e0245839. doi: 10.1371/journal.pone.0245839 (PMC7840028; doi:10.1371/journal.pone.0245839)
Supplement: S1 Text — (DOCX) [file pone.0245839.s003.docx]

**S1 Text: Survey questionnaire in English**

Patient code____________ health facility name____________________

Date of interview______________

Interviewer name______________ signature________________

Checked by: name of supervisor/investigator______________

Section one: demographic and Socio economic characteristics of the HH

| S. No | Question | Option (Measurements) | Skip |
| --- | --- | --- | --- |
| 101. | Sex | 1. Male 2. Female |  |
| 102. | Age in Year |  |  |
| 103 | Religion | 1. Orthodox tewahido 2.muslim 3. Catholic  4. Protestant 99. Others(specify)_____ |  |
| 104 | Ethnicity | 1.Amhara 2.Tigray  3. Oromo 4.Gumuz 99. other (specify)____ |  |
| 105 | Marital status | 1. Single 2. Married 3. Separated  4. Widowed 5.Divorced 6.Others,specify___ |  |
| 106 | Educational status? | 1. Can’t read and write 2. Read and write  3. Grade 1-8 4. Grade 9-12  5. Diploma and Above |  |
| 107 | Your major/ main occupation | 1. Unemployed 2.Government employed  3. NGO Employed 4. Retired 5. Student  6. House wife 7. Merchant 8. Private business 9. Farmer 99. Others specify | If it is not 9, skip to 109 |
| 108 | Role in household | 1. Father 2. Motherhood 3. Child 99. Other |  |
| 109 | Number of Family members in the household |  |  |

| 110 | Number of children <5 years |  |  |
| --- | --- | --- | --- |
| 111 | age of older household member | _____years |  |
| 112 | What is your average monthly income? | _____Birr |  |
| 113 | On average how much is monthly income of your household? | ________Birr |  |
| 114 | Total annual income of the household? | ________ETB |  |
| 115 | Does the Household have farm land? | Yes; Size in hectares_____  No |  |
| 116 | Do the household have any  annual income earned as a result of sales of: | Teff; 1. Yes (in quintal) _______ 0. No  Maize; (1. Yes (in quintal) _______0. No  Khat;1. Yes (in Kg) __________ 0. No  Grains; 1. Yes (in quintal) _______0. No  Fruit; 1. Yes (in Kg) _________ 0. No  Other specify (in quintal)____ |  |
| 117 | What is the source of income in the past 12 months? | 1. Permanent job 2. Agricultural product  3. Livestock breeding 4. Others(specify)____ |  |
| 118 | Which of these animals do this household own? | 1. Milk cows, oxen or bulls? 1. Yes, how many___0. No  2. Goats? 1. Yes, how many________ 0. No  3. Sheep? 1. Yes, how many________ 0. No  4. Chickens? 1. Yes, how many________ 0. No  5. Beehives 1. Yes, how many_________ 0. No  6. Horse/mule /donkey 1. Yes, how many____ 2.No  7. Other(specify) ______________ |  |
| 119 | Does the family have | Functioning radio/tape 1.Yes 0. No  Functioning TV 1.Yes 0. No  Cotton/sponge/spring mattress? 1. Yes 0. No  Bed 1.Yes 0. No |  |
| 120 | What is the type of roof of the house? | Thatch roof  Corrugated sheet  Other (specify)________ |  |
| 121 | What is the wall of your residence house made of | Wood with Mud  Concrete  Other (specify)___________ |  |
| 122 | How many bed rooms do you have in the household? | ____rooms |  |
| 123 | Do you have separate rooms for cattle? | 1.yes 0.No |  |
| 124 | Do you have kitchen? | 1.yes 0.No |  |
| 125 | Does the household have electricity? | 1. Yes 0. No |  |
| 126 | What is the type of fuel for cooking? | 1. Wood 2. Charcoal 3. Biogas  4. Kerosin 5. Electricity |  |
| 127 | Is this house your own? | 1.yes 0.No |  |
| 128 | What kind of latrine does your family have? | 1. None 2.Traditional latrine 3.VIP  4.Other (specify)___________ |  |
| 129 | What is source of water for this household use? | 1. River 2. Spring  3. Hand well 4. pipe water |  |

**Section two: clinical characteristics of diabetic mellitus and related issues**

| S.No | Question | Option | Skip |
| --- | --- | --- | --- |
| 201 | Duration of follow up? | years |  |
| 202 | What type of diabetic case do you have? | 1. Type 1 2. Type 2 3. I don’t know |  |
| 203 | How frequently on average do you visit a health facility monthly? | 1.____times/month  2. Other (specify)____ |  |
| 204 | On average how many days did spent/visit | ___days |  |
| 205 | During checkup, on average, how long do you wait at reception to see a doctor? | ------hours |  |
| 206 | Do you have any other comorbid illness in the last one year? | 1.yes  2.no |  |
| 207 | If yes in question no 205, how much it costs on average? | --------Eth birr |  |
| 208 | Do you have taken any prevention action to minimize complication? | 1. Yes  2. No | If 2 skip  to 210 |
| 209 | If yes Q 208 above, What type of prevention does have you taken? How much does it cost per months? | 1. Control of feeding habit birr  2. Regular exercise birr  3. Other birr  4. Total birr |  |
| 210 | Do you have worries because of your DMs conditions? | 1. Yes  2. No | If 2 Skip  to 301 |
| 211 | If yes in above Q 209, Why do you worries? | Illness of the diseases  Cost of medication  3. Limited feeding, social interactions  4. Others |  |
| 212 | If yes in Q 209, how much you are affected? | 1. Very strongly 2. Strongly  3. medium 4.Fairly 5.Rarely |  |

**Section three: cost of treatment**

| 301 | How frequently do you take Regular medications and laboratory test? | 1. ____________medication  2._____________Laboratory test |  |
| --- | --- | --- | --- |
| 302 | What kinds of medications and Laboratory test do you use? What is your single bought or regular medication cost and for how long do you use it? | 1.Laboratory tests birr for time  2.Insulin birr for times  3.Insulin syringes birr for times  4.Oral anti diabetic agent __birr for times  medical card ____for____times  6. Others(specify) birr for times  7. Total average cost |  |
| 303 | Mainly/regularly from where do You get the medications and other services? | 1. Governmental health facility 2. Private health facility 3. NGOs 4. DMs Associations 5. Others/specify |  |
| 304 | During your regular follow up to Health facilities do you use services like cafeteria? | 1.Yes  2.No | If 2 skip  to 306 |
| 305 | If yes in Q 304, how much do you and your caregiver costs on average per visit? | _______Etbirr |  |
| 206 | How much did you and your caregiver averagely pay for lodging per visit? | _______ Etbirr |  |
| 307 | What is your financial source for diabetic mellitus medications? | 1. Government frees 2.self 4. Family/relatives 99 Others_____ |  |

**Section four: loss of work days**

| S. No | Question | option | Skip |
| --- | --- | --- | --- |
| 401 | Because of DMs do you have stopped going to school/ work | 1. Yes  2. No | If 2 skip  to 403 |
| 402 | If yes in Q 401, for how many days on average were you absent? | 1._______days from school  2. _______days from work |  |
| 403 | How many days did you come to health facilities for follow up on average monthly? | ________days |  |
| 404 | Has someone come with you for follow up? | 1. Yes  2. No | If 2 skip  to 407 |
| 405 | If yes in Q 404, How many caregivers were with you? |  |  |
| 406 | If yes in Q 404, how many days your caregiver with you? | ______Days |  |
| 407 | Occupation and average monthly income of care giver | 1.Occupation____  2.Average monthly income___ |  |
| 407 | Do you had household illness or in care? | 1. Yes  2. No | If 2 skip  to 409 |
| 408 | If yes in Q above how many days? | ______days |  |
| 409 | Is there any one give you care in house hold in last 12 months when you get sick? | 1. Yes  2. No |  |
| 410 | How many care givers |  |  |
| 411 | If yes in Q 409, how many days they have had? | ______Days |  |
| 412 | Occupation and average monthly income of caregiver |  |  |

**Section five : cost of transportation**

| S. No | Question | option |  |
| --- | --- | --- | --- |
| 501 | What is the means of transport to get to the health facility during your follow up? | 1. On feet 2. car  99, other , specify |  |
| 502 | How long does it take to health facility during follow up? | 1. ______minutes  2. ______ km |  |
| 503 | How many trips do you have had per month averagely? |  |  |
| 504 | What is your single trip total cost of transportation? | ______birr |  |
| 505 | Has anyone from your family/friends looked after you when you visited hospital? | 1. Yes  2. No | If 2 skip  to 507 |
| 506 | If yes in Q 505, what is your caregiver single trip total transportation cost? | _____birr |  |
| 507 | In last 12 months, do you have had an emergency transport ? | 1.Yes  2. No |  |
| 508 | If yes in Q 507, how many times do you have had, how much was average transport costs per single visits? | 1. _______times  2. _________Birr |  |

**Section six: Coping Costs/strategies**

| No | Question | Answer | Skip |
| --- | --- | --- | --- |
| 601 | Where did you get the money from to cover the above mentioned costs | Own money (salary, savings)  I borrow money/loan  I sold my assets  My family/relatives support  99. Others_______ | If 3 skip 706. |
| 602 | If you borrowed to cover your Medication cost, how much money did you borrow? | ________birr |  |
| 603 | From whom did you borrow? | 1. Family 2. Cooperative  3 Neighbors/friends 4. Private bank 99. Others |  |
| 604 | If you sold your property for your treatment or to repay your loan, what type of property you sold or you have plan to sale? | 1. Household item 2. Jewelry  3. Vehicle 4. House  5. Land 99. Others |  |
