# Supplementary material for: Are Ethiopian diabetic patients protected from financial hardship?
Source: PLoS One. 2021 Jan 27;16(1):e0245839. doi: 10.1371/journal.pone.0245839 (PMC7840028; doi:10.1371/journal.pone.0245839)
Supplement: S2 Text — (DOCX) [file pone.0245839.s004.docx]

S2 Text: Local language version questionnaire (Amharic language)

ባህር ዳር ዩኒቨርሲቲ ህክምናና ጤና ኮሌጅ የህብረተሰብ ጤና አጠባበቅ ተቋም ለስኳር ህመም ታካሚዎች የተዘጋጀ መጠየቅ

የፈቃደኝነት መግለጫ ቅፅ

እንደምን አደሩ /ምንዋሉ/ ስሜ ---------------------------------------ይባላል፡፡ ዛሬ እዚህ የተገኘሁት በድህረ-ምረቃ ፕሮግራም ስር በባህር ዳር ከተማ ባሉ የተመረጡ ሆስፒታሎች በሚከታተሉ የስኳር ህምምተኞች እና ቤተሰቦቻቸው ላይ የስኳር ህሙም የሚያመጣውን ኢኮኖሚያዊ ጫና ለይ ለሚደረግ ጥናት መረጃ ለመሰብሰብ ነዉ፡፡ ጥናቱ የሚካሄደው ባህር ዳር ዩኒቨርሲቲ ህክምናና ጤና ኮሌጅ የህብረተሰብ ጤና አጠባበቅ ተቋም ትምህርት ክፍል የድህረ-ምረቃ ተማሪ በሆኑት ዶር ገብረማሪያም ጌታነህ ነው፡፡ ጥናቱ የታሰበለትን ዓላማ ያሳካ ዘንድ የእርስዎ በፍቃደኝነት ላይ የተመሰረተ ቀና ትብብር ያስፈጋል. ስምዎን ወይም የመለያ ቁጥርዎን በቅፅ ላይ መፃፍ አይጠበቅብዎትም፡፡ መልሶችዎ ሙሉ በሙሉ በሚስጥር የሚያዙ ይሆናሉ፡፡ በመጠይቁ የመሳተፍ አሊያም ያለመሳተፍ ሙሉ መብት የእርስዎ ነው፡፡በመጠይቁ በመሳተፈወ የተለየ የሚያገኙት ጥቅም አይኖርም፡፡

መጠይቁን ሞልተዉ ለመጨረስ ከ 20 ደቂቃ በላይ አይፈጅበዎትም፡፡ማንኛውም ዓይነት የሚሰጡን መረጃ/ምላሽ በሚስጥር የምንጠብቅ ሲሆን የዚህ ጥናት መሳካት እርሶ በሚሰጡን ትክክለኛ መልስ ላይ የተመሰረተ በመሆኑ እባክዎን ይህንን መጠይቅ በሚገባ ካነበቡ በኃላ በአግባቡ /ትክክለኛውን ምላሽ ይስጡ፡፡

ማንኛውን ጥያቄ ያልገባዎት ሀሳብ ካለ ዶር ገብረማሪያም ጌታነህን በስልክ ቁጥር

0929267153 ወይም

በኢሜል [getanehgebremariam@gmail.com](mailto:getanehgebremariam@gmail.com) መጠይቅ ይችላለ፡፡

ከተስማሙ መጠይቁን ቀጥሉ

ካልተስማሙ ይለፉ

መጠይቅ

የተሳታፊ ግለሰብ መለያ ኮድ--------- የጤና ተቋሙ ስም-------------- ጥያቄዉ የተጠየቀበትቀን------

የጠያቂዉ ስም ------------- ፊርማ ---------

የተቆጣጣርው/ዋ ስም-----------------------ፊርማ---------------

ክፍል አንድ ማህበራዊ ኢኮኖሚያዊ እና አጠቃላይ የግለሰብ መረጃ

| ተ*.*ቁ | ጥያቄ | አማራጭ | እለፍ |
| --- | --- | --- | --- |
| 101 | ጾታ | 1. ወንድ 2. ሴት |  |
| 102 | ዕድሜ |  |  |
| 103 | ሀይማኖት | 1. ኦርቶዶክስ ተዋህዶ 2. ሙስሊም  3. ካቶሊክ 4. ፕሮቴስታንት 5. ሌላ ካለ ይገለፅ |  |
| 104 | ብሄር | 1. አማራ 2. ኦሮሞ  3. ትግራይ 4. ጉሙዝ 5. ሌላ ካለ ይገለፅ |  |
| 105 | የጋብቻ ሁኔታ | 1. ያላገባ 2. ያገባ/አብሮ የሚኖሩ 3. ተለያይተዉ የሚኖሩ  4. ባል/ሚስት የሞተበት 5. የተፋታ/ች 6. ሌላ ካለ ይገለፅ |  |
| 106 | የትምህርት ሁኔታ | 1. ማንበብና መጻፍ የማይችል 2. ማንበብና መጻፍ የሚችል  3. ከ1ኛ-8ኛ ክፍል 4. ከ9ኛ-12ኛ ክፍል 5. ዲፕሎማና ከዚያ በሊይ |  |
| 107 | ዋና ስራዎ ምንድን ነዉ? | 1. ስራ የሌለዉ 2. የመንግስት ተቀጣሪ 3. መንግስታዊ ልሆነ ተቀጣሪ  4. ጡረተኛ 5 . ተማሪ 6. የቤትእመቤት 7. ነጋዴ 8. የግል ስራ 9. ግብርና ስራ 99. ሌላ ካለ ይገለፅ | መልስወ1፣3፣4፣5፣6፣7 ከሆነ ወደ ጥያቄ 10ይሂዱ |
| 108 | ቤት ውስጥ ያለዎት ኃሊፊነት? | 1. አባት 2. እናት 3. ሌጅ 4. ሌላ |  |
| 109 | በቤተሰብዎ ዉስጥ የሚገኙ ሰዎች ብዛት |  |  |
| 110 | ከ5 ዓመት በታች ያሉ ህፃናት ብዛት |  |  |
| 111 | ትልቁ የቤተሰብ አባል እድሜ ስንት ነዉ; |  |  |
| 112 | የእርስዎ አማካኝ ወርሃዊ ገቢ ስንት ነዉ? |  |  |
| 113 | የቤተሰቡ አማካኝ ወርሃዊ ገቢ ስንት ነዉ? |  |  |
| 114 | የቤተሰቡ አጠቃላይ አመታዊ ገቢ ስንት ነዉ? |  |  |
| 115 | ቤተሰቡ የእርሻ ቦታ አላችሁ | 1.አወ ፡ምን ያክል ሄክታር  2.የለንም |  |
| 116 | ቤተሰቡ ከሚከተሉት ሰብሎች የምታገኙት አመታዊ ገቢ አለ | 1.ጤፍ 1.አወ (በኩንታል___________0.የለም  2.በቆሎ 1.አወ (በኩንታል_____________0.የለም  3.ጫት 1.አወ (በኪ.ግ________________0.የለም  4.ጥራጥሬ1.አወ (በኩንታል_____________0.የለም  5.ፍራፍሬ 1.አወ (በኪ.ግ)_______________0.የለም  99. ሌላ ካለ ይገለፅ (በኩንታል)_____________ |  |
| 117 | የቤተሰቡ ዋና የገቢ ምንጭ ምንድን ነው | 1.ቁዋሚ ስራ 2. የግብርና ዉጤት  3. እንስሳት እርባታ 99. ሌላ ካለ ይገለፅ |  |
| 118 | ቤተሰቡ ከሚከተሉት አንስሳት የትኞቹ ይኖሩታል | 1. የወተት ላም፣በሬ 1.አለን፣ስንት ናቸው_______________0. የለንም  2. ፍየል 1.አለን፣ስንት ናቸው _______________0. የለንም  3. በግ 1.አለን፣ስንት ናቸው _______________0. የለንም  4. ዶሮ 1.አለን፣ስንት ናቸው________________0. የለንም  5. ንብ 1.አለን፣ስንት ናቸው_______________ 0. የለንም  6.ፈረስ/በቅሎ/አህያ 1.አለን፣ስንት ናቸው_______________ 0. የለንም  99. ሌላ ካለ ይገለፅ |  |
| 119 | ከሚከተሉት ውስጥ ቤተሰቡ ምን አለው | 1. ራዲዎ/ቴፕ 1. አወ 0. የለም  2. ቴሌቪዥን 1. አወ 0. የለም  3. ጥጥ/ስፖንጅ ፍራሽ 1. አወ 0. የለም  4. አልጋ 1. አወ 0. የለም |  |
| 120 | የቤታችሁ ጣራ የተሰራው ከምንድን ነው | 1.ከሳር 2.ከቆርቆሮ 99. ሌላ ካለ ይገለፅ |  |
| 121 | የቤታችሁ ግድግዳ ምንድን ነው | 1. እንጨት እና ጭቃ 2.ብሎኬት 99. ሌላ ካለ ይገለፅ |  |
| 122 | ቤታችሁ ስንት የኣልጋ ክፍሎች አሉት | ____________ |  |
| 123 | የእንስሳት ማደሪያ የተለየ ክፍል አላችሁ | 1.አወ 0.የለም |  |
| 124 | ኩሽና ቤት አላችሁ | 1.አወ 0.የለም |  |
| 125 | ኤሌክትሪክ መብራት አላችሁ | 1.አወ 0.የለም |  |
| 126 | ምግብ ለማብሰል የምትጠቀሙት ምንድን ነው | 1.እንጨት 2.ከሰል 3 ባዮጋዝ 4.ናፍጣ 5. ኤሌክትሪክ |  |
| 127 | የምትኖሩበት ቤት የራሳችሁ ነው | 1. አወ 0. አይደለም |  |
| 128 | ምን አይነት ሽንት ቤት ነው ያላችሁ | 1. የለንም 2.ባህላዊ 3.ቪአይፒ 99. ሌላ ካለ ይገለፅ |  |
| 129 | የምትጠቀሙት ምን አይነት ውሃ ነው | 1. ወንዝ 2. ምንጭ 3.ጉድድ 4.ቧንቧ |  |

ክፍል ሁለት: የስኳር ህመም ዓይነትና የግለሰቦች ድርሻ

| ተ.ቁ | ጥያቄ | አማራጭ | እለፍ |
| --- | --- | --- | --- |
| 201 | ለምን ያህል ጊዜ ነዉ የስኳር ህመም ክትትል ያደረጉት? | _______________ዓመት |  |
| 202 | ምን አይነት የስኳር ህመም ነዉ ያለብዎት? | 1. ታይፕ 1 2. ታይፕ 2 |  |
| 203 | በአማካኝ በወር ስንት ጊዜ ለክትትል ይመጣሉ? | 1 በወር----ጊዜ 99. ሌላ ካለ ይገለፅ |  |
| 204 | በአማካኝ አንድ ከትትል ምን ያክል ይጨርስብሃል |  |  |
| 205 | ለክትትል ሲመጡ ሀኪሙን ለማገኘት በአማካይ ምን ያህል ጊዜ/ሰዓት ይጠብቃሉ ? | _____________ሰዓት |  |
| 206 | ባለፈው አንድ አመት ሌላ ተዛማጅ ህመም አሞዎት ያውቃል፣ምን |  |  |
| 207 | አሞዎት ከነበር ለህክምና ምን ያህል ወጭ አስወጠዎት | ________________ብር |  |
| 208 | የስኳር ህመምዎ እንዳይባባስ የሚወስዱት ወይም የሚሰሩት የመከላከያ ተግባር አለ? | 1. አዎ  2. የለም | መልሶ 2 ከሆነ ወደ ጥያቄ  210 ይሂዱ |
| 209 | ከላይ ለቀረበው ጥያቄ መሌሶ አዎ ከሆነ፣ ምን ዓይነት ተግባር ነው የሚያከናውኑት?ለዚህስ ምን ያህል ወጪ በወር ያወጣሉ? | 1. አመጋገብ ማስተካከል ብር  2. የአካል ብቃት እንቅስቃሴ ብር  3. ሌላ ካለ ብር  4. ድምር ብር |  |
| 210 | ከስኳር ህመምዎ ጋር በተያያዘ ሀሳብ/ጫና/ጭንቀት አለብዎት? | 1. አዎ  2. የለብኝም | መልሶ 2 ከሆነ ወደ ጥያቄ  301 ይሂዱ |
| 211 | ከስኳር ህመሞ ጋር በተያያዘ ምንድነው ጭንቀቶዎት ስሜቶዎን የሚጎዳው/የሚነካው፣ የሚያሳስቦት ነገር ምንጭ?(ከአንድ በላይ መልስ ይቻላል) | 1. የበሽታው ህመም 2. የህክምና ወጪ  3.የአመጋገብ ሁኔታ፤ እርበርስ ግንኙነት  4. ሌላ ካለ |  |
| 212 | ከላይ ለቀረበዉ ጥያቄ መሌስዎ አዎ ከሆነ ምን ያህሌ ነዉ ጫናው? | 1. በጣም ከፍተኛ 4. መጠነኛ  2. ከፍተኛ 5. አነስተኛ  3. መካከለኛ |  |

ክፍል ሶስት: የስኳር ህክምና ወጪ

| ተ.ቁ | ጥያቄ | አማራጭ | እለፍ |
| --- | --- | --- | --- |
| 301 | በየምን ያህል ጊዜ ነው መድሀኒት እና ላቦራቶሪ ምርመራ የሚወስዱት? | 1. __________የህክምና መድሃኒ  2. _______ምርመራ/ላ ቦራቶሪቴስት |  |
| 302 | ምን ዓይነት ህክምና ነው የሚወስዱት፤ የአንድ ጊዜ ህክምና ወጪዎት ምን ያህል ነው፣ ለምን ያህል ጊዜ ነው የሚጠቀሙት? | 1.የላቦራቶሪ ምርመራ ብር ጊዜ  2.እንሱሊን ብር ጊዜ  3.የእንሱሊን ሲርንጂ ብር ጊዜ  4.በአፍ የሚወሰድ መድሃኒት ብር ጊዜ  5.ለካርድ__________ብር____________ጊዜ  6. ሌላ ካለ _______ ብር  ጊዜ  7. በአጠቃሊይ ብር ለጊዜ |  |
| 303 | አብዛኛውን ጊዜ የስኳር ህክምና አገሌግልቶችን የሚያገኙትከየት ነው ? | 1. ከመንግስት ጤና ተቋም 2. ከግሌ ጤና ተቋም  3. መንግስታዊ ካሌሆነ ተቋም 4. ከማህበር/ከስኳር  5. ከሌላ ቦታ-------- |  |
| 304 | ባለፉት ስድስት ወራት ለክትትል ጤና ተቋም በሚመላለሱበት ወቅት ለምግብና ለመስተንግዶ ነገሮች ወጪ አውጥተው ነበር? | 1. አዎ  2. የለም | መልሶ 2 ከሆነ ወደ ጥያቄ 306 ይሂዱ |
| 305 | መልሶ አዎ ከሆነ እርሶና አብሮት የነበረው ሰው በአማካይ በአንድ ደርሶ መልስ ጉብኝት ምግብ ምን ያህል ወጪ አወጣችሁ | _____________ብር |  |
| 306 | በአማካኝ በአንድ ክትትል ለአልጋ/ማርፊያ እርሰወ እናአብሮት የነበረው ሰው ምን ያክል ዎጭ ታዎታላችሁ |  |  |
| 307 | ለስኳር ህክምና ዋና የገንዘብ ምንጭዎት ከየት ነዉ?(ከአንድ በላይ መልስ ይቻላል) | 1. ነፃ ህክምና 2. ኢንሹራንስ  3. ከግል ኪስ ወጪ 4. ከቤተሰብ/ከጓደኞች  5. ሌላ የወጪ ምንጭ ካለ ይጠቀስ---- |  |

ክፍል አራት: ለህክምና የዋሉ ቀናት

| ተ*.*ቁ | ጥያቄ | አማራጭ | እለፍ |
| --- | --- | --- | --- |
| 401 | ባለፉት ስድስት ወራት ከስኳር ህመሙ ጋር በተያያዘ ት/ቤት ወይም ስራ የቀሩበት ቀናት ነበርዎት? | 1. አዎ  2. የለም | መልሶ 2 ከሆነ ወደ ጥያቄ 403 ይሂዱ |
| 402 | በጥያቄ ቁጥር 401 መልሶ አዎ ከሆነ በአጠቃላይ ባለፉት ስድስት ወራት ምን ያህል ቀን ከት/ቤት ወይም ከስራ ቀሩ? | 1.__________ቀናት ከት/ቤት  2.___________ቀናት ከስራ |  |
| 403 | ባለፉት ስድስት ወራት እርስዎ ለክትትል ወደ ጤና ተቋም ሲመጡ ምን ያህሌ ቀናትን ተጠቀሙ? | ___________ቀን |  |
| 404 | ባለፉት ስድስት ወራት ከእርሶ ጋር ጤና ተቋም የሚመጡ ሰዎች ወይም አስታማሚዎች ነበርዎት? | 1. አዎ  2. አይ | መልሶ 2 ከሆነ ወደ ጥያቄ 407 ይሂዱ |
| 405 | መልሶ አዎ ከሆነ ከእርሶ ጋር ጤና ተቋም የሚመጡ ሰዎች ወይም አስታማሚዎች ቁጥራቸዉ ስንት ነው? | _______ |  |
| 406 | መልሶ አዎ ከሆነ ከእርሶ ጋር ጤና ተቋም የሚመጡ ሰዎች ወይም አስታማሚዎች ምን ያህል ቀናትን ቆዩ? | _____________ቀን |  |
| 407 | የአስታማሚወ ስራ እና አማካኝ ወራዊ ገቢ ስንት ይሆናል | 1. ስራ___________________________  2.አማካኝ ወራዊ ገቢ_____________________ |  |
| 408 | ባለፉት ስድስት ወራት ቤት ውስጥ ተኝተው/ከአቅም በላይ ሆኖቦዎት ያውቃል? | 1. አዎ 2. የለም | መልሶ 2 ከሆነ ወደ ጥያቄ 411 ይሂዱ |
| 409 | መልሶ አዎ ከሆነ ባለፉት ስድስት ወራት ምን ያህል ቀን ይሆናል? | _________________ቀን |  |
| 410 | ባለፉት ስድስት ወራት ቤት ውስጥ ተኝተው/ከአቅም በላይ ሆኖቦዎት ድጋፍና እንክበካቤ የሚያደርግሌዎት ሰው ነበረ? | 1. አዎ  2. የለም | መልሶ 2 ከሆነ ወደ ጥያቄ 411 ይሂዱ |
| 411 | ስንት አስታማሚዎች እንክብካቤ አደረጉለዎት | _________ |  |
| 412 | በጥያቄ ቁጥር 407 መልሶ አዎ ከሆነ ለምን ያህል ቀን እንክብካቤ ተደረገለዎት? | _______________ቀን |  |
| 413 | የአስታማሚወ ስራ እና አማካኝ ወራዊ ገቢ ስንት ይሆናል | 1. ስራ  2.አማካኝ ወራዊ ገቢ |  |

ክፍል አምስት: የመጓጓዣ ወጪ

| ተ*.*ቁ | ጥያቄ | አማራጭ | እለፍ |
| --- | --- | --- | --- |
| 501 | ክትትል ወደ ሚያደርጉበት የጤና ተቋም ሲሄዱ ምን አይነት የመጓጓዣ ዘዴ ይጠቀማለ? | 1. እግር 2.በመኪና  3. ሌላ ዘዴ ካለ ይጠቀስ --------- |  |
| 502 | ክትትል ወደሚያደርጉበት የጤና ተቋም ለመድረስ በአማካይ ምን ያህል ጊዜ ይፈጅብዎታል ርቀቱስ ምን ያህል ኪ.ሜ ነው? | 1. __________ደቂቃ  2. ኪ.ሜ |  |
| 503 | ባለፉት ስድስት ወራት ለክትትሌ ምን ያህል የደርሶ መሌስ ጉዞ አደረጉ? |  |  |
| 504 | የአንድ ጉዞ ደርሶ መልስ የመጓጓዣ ወጪዎት ምን ያህል ነው? | ____________ብር |  |
| 505 | ለክትትል ሲመጡ ከእርሶ ጋር ወደ ጤና ተቋም የሚመጣ ሰው አለ? | 1. አዎ  2.የለም | መልሶ 2 ከሆነ ወደ ጥያቄ 507 ይሂዱ |
| 506 | የእርሶ ድጋፍ ሰጪ ሰው ደርሶ መልስ የመጓጓዣ ወጪው ስንት ነው? | __________ብር |  |
| 507 | ላለፉት ስድስት ወራት ለድንገተኛ ህክምና የመጓጓዣ አገሌግልት ተጠቅመው ነበር ? | 1.አዎ  2. የለም | መልሶ 2 ከሆነ ወደ ጥያቄ 601 ይሂዱ |
| 508 | መልሶ አዎ ከሆነ ላለፉት ስድስት ወራት ምን ያህሌልጊዜ ድንገተኛ ህክምና አደረጉ፤ የአንድ ጊዜ የጉዞ አማካይ ወጪዎ ምን ያህል ነበር? | 1._____________ጊዜ  2 የአንድ ጉዞ ወጪ___________ ብር |  |

ክፍል ስድስት : የመጋለጥ ወጪ

| ተ.ቁ | ጥያቄ | መልስ |  |
| --- | --- | --- | --- |
| 601 | የስኳር በሽታ ህክምናን ለመከታተልል ወጪውን ከየት ነዉ የሚያዎጡ | 1.ከራሰ ገንዘብ (ከደሞዝ, ከቁጠባ)  2.ገንዘብ ተበድሬ/ከብድር  3.ንብረቴን ሸጬ ነው  4. ከቤተሰብ/ከጓደኛ ርዳታ ነው 99. ከሌላ | ምርጫህ1 ከሆነ ወደ ጥያቄ 701 ሁለተኛው ከሆነ ወደ ጥያቄ 703 ሦስተኛውን ከሆነ ወደ ጥያቄ 707 ተሻገር |
| 602 | ተበድረው ከሆነ ስንት ብር ተበደሩ | ___________________ብር |  |
| 603 | ከማንስ ተበድረው ታከሙ | 1. ከቤተሰብ 2. ከጎረቤት/ከጓደኛ 3. ከግል ባንክ  4. በትበብር የሚደረግ 99. ሌላ |  |
| 604 | የሰኳር ህመም ህከምና ወጪን ለመሸፈን የተሸጡ የንብረት አይነቶች ካሉ ለምሳሌ | 1. የቤት ዕቃ 2. ወርቅ 3. ተሸከርካሪ /መኪ  4. ቤት 5. መሬት 99. ሌላ |  |
