# Supplementary material for: Are Ethiopian diabetic patients protected from financial hardship?
Source: PLoS One. 2021 Jan 27;16(1):e0245839. doi: 10.1371/journal.pone.0245839 (PMC7840028; doi:10.1371/journal.pone.0245839)
Supplement: Attachment — Submitted filename: Recomandation.docx [file pone.0245839.s005.docx]

**Recommendation**

- Accept with minor revision

**Comments to the Author**

1. **Yes**
2. **Yes**
3. **Yes**
4. **Yes**
5. Yes
6. Review Comments to the Author

Financial hardship of diabetic careis the topic of this article which is a burning issue in both the global and in the Ethiopian contexts as the authors described in the introduction section of the manuscript.But as the best search of the reviewer, there is limited evidence about it in Ethiopia. The results of this study can be used as an evidence to design evidence based intervention in Ethiopia to protect diabetic patients from financial hardship, to ensure equity health care in the country. Therefore, this article is important and relevant to policy makers of Ethiopia’s health sector to accomplish Universal Health Coverage (UHC) by the year 2030.

However, the reviewer recommends the following minor revisions in the manuscript before publication.

**In the introduction section:** In the manuscript, some of the sentences are long which should be short as much as possible so that the potential readers will understand the paper easily.

- In the methods section of the manuscript, Describe your study area how many public hospitals found in Bahir Dar city administration?
- What is your sampling method? describe it clearly

**Result section**

- use appropriate punctuations in some of sentences and correct the editorials for language
- on table 1 'Amara' or 'Amhara'?
- make Table 5 clear in meaning and drawing as well
