# Supplementary material for: Are Ethiopian diabetic patients protected from financial hardship?
Source: PLoS One. 2021 Jan 27;16(1):e0245839. doi: 10.1371/journal.pone.0245839 (PMC7840028; doi:10.1371/journal.pone.0245839)
Supplement: Attachment — Submitted filename: comments to the Author.docx [file pone.0245839.s006.docx]

**Comments to the Author**

**Abstract**

- line # 32 add "of" Forty percent of nonfood threshold level was
- line #40&41 had statistically significant association with the catastrophic diabetic care expenditure.

**Method**

- Describe your study area how many public hospitals found in Bahir Dar city administration?
- What is your sampling method? describe it clearly

**Result**

- use appropriate punctuations in some of sentences and correct the editorials for language
- on table 1 'Amara' or 'Amhara'?
- make Table 5 clear in meaning and drawing as well
