# Supplementary material for: Are Ethiopian diabetic patients protected from financial hardship?
Source: PLoS One. 2021 Jan 27;16(1):e0245839. doi: 10.1371/journal.pone.0245839 (PMC7840028; doi:10.1371/journal.pone.0245839)
Supplement: Attachment — Submitted filename: Author-responses letter.docx [file pone.0245839.s007.docx]

Dear reviewers and editor,

Thank you so much for your constructive comments to improve our manuscript.

Below are our point by point responses for the comments

**Responses for the academic editor**

1. We have adhered to the requirements of PLOS ONE's style as much as possible**.**
2. We, the authors, and our colleague (Dr.Walelign Kindie, Email: [walelignkindie2@gmail.com](mailto:walelignkindie2@gmail.com), an academic staff of Bahir Dar University, Tel: +2519 38883386) thoroughly copyedited the manuscript for language usage, spelling, and grammar.
3. We, the authors, submitted the local language, Amharic, and English versions of questionnaire tools as additional files.
4. We have conducted pretest before survey as stated on page 6 line number 106 in the manuscript. The purpose of the pretest was to ensure that the understandability and clearness of the questionnaire for the respondents and it was modified accordingly. We modified the flow, order, skip patterns and the allocated time for interview in the questionnaire. The questionnaire was developed based on previous validated questionnaires (standard one) that is why validation is not done for our study.

**Responses for the comments (reviewer 1)**

1. We added the response rate in the abstract section of manuscript in the result part on page 2 line number 37.
2. In the introduction section of the manuscript, long sentences have been broken down to simple, short and concise sentences as per the comments. For instance the long sentence such as, “It is a key health system objective and one of the target in the Sustainable Development Goals (SDGs), specifically SDG3, and Health Sector Transformational Plan (HSTP) of Ethiopia” were broken down to two short and concise sentences: It is a key health system objective and one of the targets in the Sustainable Development Goals (SDGs), specifically SDG3. Financial risk protection is also the target of Health Sector Transformational Plan (HSTP) of Ethiopia (on page 4 line numbers 54-57).
3. In the previous manuscript ,there is empty page, it was omitted right now
4. The title of table 6 was edited and its footnote was unbold?
5. The authors added the questionnaire as supporting information in the current manuscript.

**Responses for the comments (reviewer 2)**

1. We used pronoun ‘we’ as a doer as the reviewer has commented on the manuscript. Even though, the interest is expressing on what has been done, to make it clear the doer is also important as per the norm of scientific writing.
2. In the conclusion part of the abstract we stated in such a way that economic burden of DM care is stronger among the less privileged populations: the less educated, the poorest and unemployed as per the recommendation of the reviewer on page 3 line numbers 43-44.
3. We edited lengthy sentences to make it simple and clear throughout the manuscript.
4. In the methods part the phrase “institutional based “was used to indicate the type of study design that we used to answer our research question. The readers (those who are not expertise in epidemiology) might not understand the type of study design by reading the setting of the study as reviewer stated.
5. We, the authors, included patients who are not CBHI enrolled as stated on page 6 line numbers 91-93 in the manuscript.
6. We, the authors, mentioned (on page 6 line numbers 100-102) how many samples we took from how many populations in both of the hospitals. We recruited 325 diabetic patients from Felege Hiwot comprehensive specialized hospital and 97 diabetic patients from Addis Alem primary hospital by using simple random sampling method with computer generated random number.
7. We, the authors, replaced health expenditure to expenditure of diabetic care in the measuring catastrophic health expenditure part of the manuscript. Moreover, we used DMxi instead of Ti. - Expenditure of the ith patient to receive care for DM. All formulas were edited as per the comments of reviewer.
8. We used a threshold of 40% nonfood expenditure for analytical analyze, however, we also reported the results at different recommended levels: varied between 10 and 40% of threshold level.
9. We elaborated more about the overshoot in the current version.
10. In the result part, we reported the monthly income in the table in the previous version. As per the comment of the reviewer, we have stated the mean monthly income in the current version of the manuscript on page 12 line numbers 207-208.
11. In table 1- the reviewer recommended that “better to cancel the variable ’Sex’. Since the males are reported in the text the reader understands that the other share is for females. The same is true for Religion and Residence. Narrate the dichotomy variables in the text and avoid including it in the table. This helps the table to be smart”. However, if we cancel these important variables, the table may not be self-explanatory or self-contained that leads to ambiguity due to the incomplete information in the table.
12. We have mentioned how the mean monthly expenditures and costs were calculated in the method of costing part starting from on page 7 line number 119.
13. Regarding to table 3 in the result part, the table informs the readers the proportion of catastrophic diabetic care expenditure and impoverishment due to diabetic care in each category, more specifically those variable not reported in the regression table. Hence, it adds value for the readers.
14. We have edited and corrected the discussion as per the recommendation of the reviewer’s comments on page 23 line numbers: 334,336 and 340.
15. In conclusion part, we have specified the last sentence as per the reviewer’s comment on page 25 line numbers 372 and 373.
16. We have edited the references list in the manuscript.

**Responses for the comments (reviewer 3)**

1. In the introduction section, we edited the long sentences in the current version of abstract.
2. In the method section, we described about the study area in which how many public hospitals found in Bahir Dar city administration on pages 5&6 line numbers 85-90.
3. Regarding to sampling method, we stated about it on page 6 line number 99-102.
4. In the result part, we and our colleague have copy edited the whole manuscript including result part.
